# Interfacial Engineering and Photon Downshifting of CsPbBr_3_ Nanocrystals for Efficient, Stable, and Colorful Vapor Phase Perovskite Solar Cells

**DOI:** 10.1002/advs.201802046

**Published:** 2019-04-20

**Authors:** Cong Chen, Yanjie Wu, Le Liu, Yanbo Gao, Xinfu Chen, Wenbo Bi, Xu Chen, Dali Liu, Qilin Dai, Hongwei Song

**Affiliations:** ^1^ State Key Laboratory on Integrated Optoelectronics College of Electronic Science and Engineering Jilin University 2699 Qianjin Street Changchun 130012 P. R. China; ^2^ Department of Chemistry Physics, and Atmospheric Sciences Jackson State University Jackson MS 39217 USA

**Keywords:** colorful, downshifting, perovskite solar cells, stability, vapor phase

## Abstract

Photovoltaic devices employing lead halide perovskites as the photoactive layer have attracted enormous attention due to their commercialization potential. Yet, there exists challenges on the way to the practical use of perovskite solar cells (PSCs), such as light stability and current–voltage (*J*–*V* ) hysteresis. Inorganic perovskite nanocrystals (IPNCs) are promising candidates for high‐performance photovoltaic devices due to their simple synthesis methods, tunable bandgap, and efficient photon downshifting effect for ultraviolet (UV) light blocking and conversion. In this work, CsPbBr_3_ IPNCs modification could give rise to the vapor phase and solution‐processed PSCs with a power conversion efficiency (PCE) of 16.4% and 20.8%, respectively, increased by 11.6% and 5.6% compared to the control devices for more efficient UV utilization and carrier recombination suppression. As far as is known, 11.6% is the most effective enhanced factor for PSCs based on photon downshifting effect inside of devices. The CsPbBr_3_ layer could also significantly retard light‐induced degradation, leading to the lifetime over 100 h under UV illumination for PSCs. Additionally, the modified PSCs exhibit weak hysteresis and multiple colors of fluorescence. These results shed light on the future design of combining a photon downshifting layer and carrier interfacial modification layer in the applications of perovskite optoelectronic devices.

## 1 Introduction

Recently, organometal halide perovskites have been considered as one of the promising photoelectric materials for solar cell application due to the advantages of high light absorption coefficient, great carrier mobility, and simple synthetic method.^1^, ^2^, ^3^ Despite perovskite solar cells (PSCs) demonstrating high performance with a certified PCE of 23.3%, they still possess poorer stability compared to Si‐ (silicon) or CIGS‐ (CuInGaSe) based photovoltaic devices.^4^, ^5^ As is well known, solar spectrum covers wavelength ranging from ultraviolet (UV) to infrared (IR) light.^6^ However, the PSCs could only utilize the visible light that accounts for only half of the solar radiation. The other half in UV and IR region is critical to design an effective strategy for high‐efficiency solar cells. In recent work, we have extended the spectrum to 1600 nm by introducing NaYF_4_: Yb^3+^, Er^3+^ photoluminescent conversion layer for converting NIR light into visible.^6^ For UV part in solar spectrum, high‐energy UV light is still an urgent problem to be solved to device degradation.^7^ UV blocking or conversion mechanism is a promising strategy to solve the light stability problem.^8^, ^9^ Therefore, light conversion of the harmful UV photons to usable visible light improves both PCE and light stability.^10^ Luminescent downshifting layers exhibit significant potential to enhance the PCE and light stability by reducing their UV degradation of PSCs.^11^, ^12^, ^13^

An ideal luminescent downshifting layer needs to have strong UV light absorption, excellent luminescence quantum yield, extraordinary transparency in the visible region, and large Stokes shift to reduce the self‐reabsorption losses. Moreover, structural simplicity, low‐cost fabrication, ambient operation, and long‐term stability are also crucial for real applications. Commonly, lanthanide‐based inorganic materials (YVO_4_:Eu^3+^, CeO_2_:Eu^3+^ or SrAl_2_O_4_:Eu^2+^, Dy^3+^) and Eu complex have the ability to absorb UV light and emit visible light used in PSCs.^14^, ^15^, ^16^, ^17^ Fluorescent carbon dots or graphene quantum dots were introduced to improve device performance by faster electron transportation.^18^ The preparation procedures of lanthanide‐ based luminescent downshifting materials are complicated, with necessary high temperature annealing process.^13^, ^19^ Exploring efficient downshifting materials and their simple preparation methods would be the focus for efficient and light‐stable PSCs. For better results, the luminescent downshifting materials must have a high fluorescence quantum yield (QE) and its optical absorption band should not overlap with the absorption of the active layer in the device. CsPbX_3_‐ (*X* = Cl, Br, I) based inorganic perovskite nanocrystals (IPNCs) exhibit ultrahigh photoluminescence quantum yields (above 90%), and the emission of CsPbX_3_ can be controlled to match well with the absorption spectrum of perovskite materials. Their superior quality in luminescence quantum yield, size‐tunable bandgap, high transparency in visible spectrum, and excellent light stability makes the CsPbX_3_ IPNCs attractive and promising in luminescent downshifting application in PSCs. When sunlight illuminates on the IPNCs, the UV light can be converted into visible light by the down‐conversion effect. Then the converted visible light can be absorbed efficiently by perovskite photoactive films. Thus, the IPNCs are deemed to be helpful in enhancing the PCE and light stability of PSCs. In this work, IPNCs were prepared using a hot‐injection method and then successfully applied to the PSCs inside and outside. Herein, we construct an appropriate IPNCs interlayer via spin‐coating method, which acts as a seed‐mediated layer for perovskite film formation, with both uniform morphology and favorable absorption capacity. In addition, IPNCs and hybrid perovskites film form a fully crystalline heterojunction, which is beneficial to minimizing the defect and trap densities. The electrical mechanism of assembled nanocrystals inside the devices was also investigated. The results reported here reveal that making full use of the UV light by CsPbX_3_ nanocrystals is beneficial for enhancing the photovoltaic performance of solar cells.

## 2 Results and Discussion

**Figure 1:**
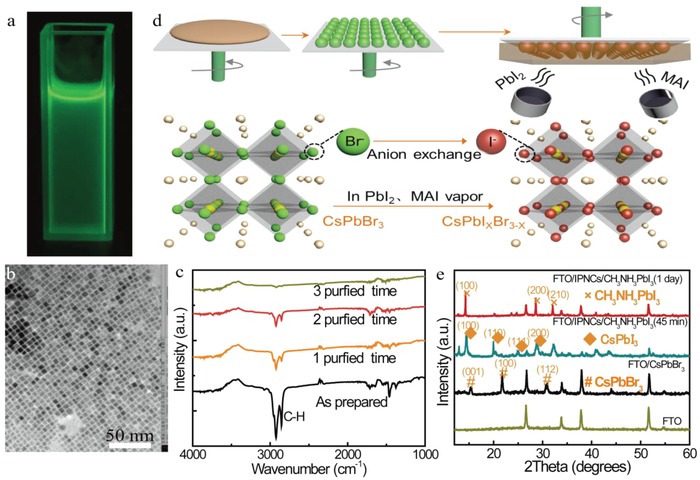
a) Photograph of the synthetized CsPbBr_3_ IPNCs under illumination of 365 nm light source. b) The TEM images of the as‐prepared nanocrystals purified by ethyl acetate. c) FTIR spectra showing the IR transmission of the as‐prepared IPNCs films and after different time ethyl acetate treated IPNCs film. d) Schematic of IPNCs‐modified PSCs by vapor deposition process with MAI and PbI_2_ source. The anion‐exchange process within the IPNCs was performed in CH_3_NH_3_I (MAI) and PbI_2_ vapor atmosphere. e) XRD patterns of the prepared samples.

The optical properties of CsPbBr_3_ photon downshifting layer was first investigated to demonstrate the feasibility of integrating the IPNCs into PSCs. More detailed information about the device fabrication can be found in the Experimental Section. In particular, the IPNCs were size‐selectively precipitated by centrifugation at a speed of 9800 rpm to ensure a narrow size distribution. For the detailed characterization, transmission electron microscopy (TEM) images in Figure S1 in the Supporting Information show cubic morphology of as‐prepared CsPbBr_3_ nanocrystals with an average size of 8 nm. Then, IPNCs with narrow size distribution were redispersed and purified in ethyl acetate before being deposited on the FTO/cp‐TiO_2_ substrate. In the purification process, the surface ligands such as oleic acid and ammonia groups can be eliminated by ethyl acetate. These ligands block the transport of photo‐generated carrier, leading to enhanced electron mobility and improved device performance.^20^ **Figure** **1**a is the photograph of the synthetized CsPbBr_3_ in ethyl acetate under UV light irradiation. The purified nanocrystals can still retain the cubic morphology for several months in ambient conditions (Figure 1b). When CsPbBr_3_ were excited by the UV light of 365 nm, the emission spectrum exhibits a narrow peak at 530 nm, with a photolumisence QY of 82.8%. Absorption and transmission curves of IPNCs are shown in Figure S2 in the Supporting Information, exhibiting the effective utilization in UV region. The absence of ≈3000 cm^−1^ and below ≈2000 cm^−1^ in Fourier‐transform infra‐red (FTIR) spectra in Figure 1c, which are attributed to C–H modes of oleylammonium, oleate, and octadecene, confirm the removal of organic ligands from the CsPbBr_3_ film by ethyl acetate with different purification time.^20^ Figure 1d shows the schematic diagram of PSC structure modified by nanocrystals, where CsPbBr_3_ was introduced into the devices. One to seven layers of purified CsPbBr_3_ were prepared on the FTO substrate by spin‐coating method to optimize the performance. Then the CH_3_NH_3_PbI_3_ films were directly deposited on top of the precoated CsPbBr_3_ layer using a solvent‐free, dual‐source vapor deposition method to avoid the dissolution of IPNCs in solvent (N,N‐dimethylformamide (DMF), dimethyl sulfoxide (DMSO) et.). Simultaneously, dual‐source vapor deposition method for CH_3_NH_3_PbI_3_ lay preparation could avoid annealing process, which is necessary for solution method.^21^, ^22^ What is worth noting is that dual‐source vapor deposition will usually sacrifice the PCE of PSCs device because it is hard to control the evaporation rate. X‐Ray Diffraction (XRD) data of CsPbBr_3_‐modified CH_3_NH_3_PbI_3_ perovskite thin films were collected in Figure 1e. For the spin‐coated CsPbBr_3_ films, the XRD patterns exhibit typical peaks of orthorhombic CsPbBr_3_ around 15° and 30°. It also shows no phase transition in the IPNCs structure during ethyl acetate purification. In order to detect changes inside the perovskite film more quickly, the thickness of CH_3_NH_3_PbI_3_ was reduced from 470 to 150 nm. XRD patterns were performed immediately after CH_3_NH_3_PbI_3_ thin film was vapor deposited. After 45 min of depositing 150 nm CH_3_NH_3_PbI_3_ film, the XRD patterns of the film exhibits a relatively strong CsPbI_3_ peak in Figure 1e, while also exhibiting a certain amount of CsPbBr_3_ peak, indicating that most of the Br ions in CsPbBr_3_ has been replaced by I ions from CH_3_NH_3_PbI_3_. Further depositing 470 nm CH_3_NH_3_PbI_3_ films on FTO/CsPbBr_3_ substrate for one day, there are no obvious CsPbBr_3_ peaks. This can be attributed to the thicknesses of FTO (570 nm) and CH_3_NH_3_PbI_3_ layer (470 nm) which are thicker than that of CsPbBr_3_ IPNCs (110 nm). Furthermore, the CsPbBr_3_ IPNCs are surrounded by CH_3_NH_3_PbI_3_ films, so the peaks of CsPbBr_3_ are hard to be detected. More importantly, most of CsPbBr_3_ has been converted into CsPbI*_x_*Br_3−_ *_x_*, and further into MA_y_Cs_1−_ *_y_*PbI*_x_*Br_3−_ *_x_* alloy, through the ion exchange process. The diffraction peaks of FTO/IPNCs/CH_3_NH_3_PbI_3_ film at 2θ = 14.2°, 28.5°, and 32.0° are assigned to (100), (200), and (210) planes of tetragonal CH_3_NH_3_PbI_3_, respectively.^23^ This result suggests that well‐crystallized perovskite films were formed on cp‐TiO_2_/IPNCs substrate, which is beneficial to improving interface contact and enhancing the PCEs.

**Figure 2:**
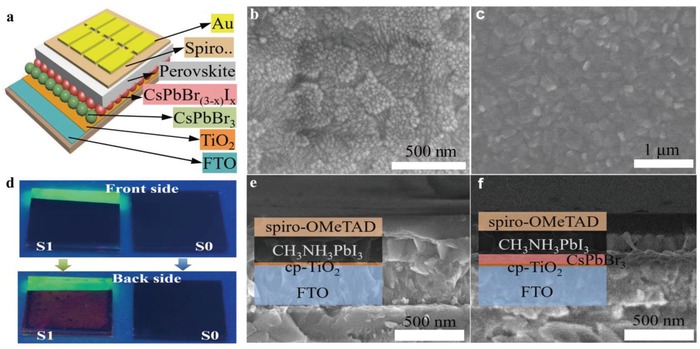
a) The device structure of CsPbBr_3_‐modified PSCs. b) The SEM morphologies of the deposited CsPbBr_3_ on FTO/cp‐TiO_2_ substrate. c) The SEM morphologies of vapor deposited CH_3_NH_3_PbI_3_ film. d) Two contrasting devices under the irradiation of UV light, S0 is a control device, while S1 is CsPbBr_3_‐modified device. Cross‐sectional SEM images of the e) control and f) CsPbBr_3_‐modified device.

**Figure** **2**a depicts the device architecture of FTO/TiO_2_/IPNCs/CH_3_NH_3_PbI_3_/spiro‐OMeTAD/Au for the CsPbBr_3_‐modified PSCs. The morphologies of the deposited IPNCs on FTO/cp‐TiO_2_ substrate were studied by scanning electronic microscopy (SEM) in Figure 2b. As is shown, the deposited IPNCs are deposited on the surface of the cp‐TiO_2_ substrate. Further increasing the amount of IPNCs layers, more nanocrystals agglomerate and the surface roughness increases obviously, as illustrated in the atomic force microscopy (AFM) images (Figure S3, Supporting Information). The root‐mean‐square (RMS) values from AFM image were calculated to be 7.8, 10.2, and 14.1 nm, revealing the enhanced surface roughness by increasing the layers. In comparison with the smooth surface of the unmodified TiO_2_ layer, the rough surface of the cp‐TiO_2_/CsPbBr_3_ layer could provide larger surface area and better contact with the hybrid perovskite layer, resulting in more effective channels for the carrier separation and collection. To give further insight in the structure of cp‐TiO_2_/CsPbBr_3_, the cross‐section images indicate that the thickness of IPNCs layer on the cp‐TiO_2_ was about 110 nm (Figure S4, Supporting Information), corresponding to five times spin‐coating of CsPbBr_3_ solution. The surface morphology of CH_3_NH_3_PbI_3_ thin film is shown in Figure 2c, which is very similar to those prepared using vapor deposition methods in previous literature.^24^, ^25^, ^26^ Figure 2d shows two devices with and without CsPbBr_3_ IPNCs modification under UV light irradiation. S0 is a control device, while the S1 is the IPNCs‐modified device. Green color fluorescence is shown for the device with IPNCs only but without CH_3_NH_3_PbI_3_ layer. Red fluorescence is observed for the device with CH_3_NH_3_PbI_3_ layer deposited on cp‐TiO_2_/ CsPbBr_3_ substrate. This can be attributed to the ions exchange of I^−^ in CH_3_NH_3_PbI_3_ with the Br^−^ in CsPbBr_3_, resulting in the formation of CsPbBr_3−_ *_x_*I*_x_* IPNCs and CH_3_NH_3_PbI_3−_ *_x_*Br*_x_*, causing the red shift of the luminescence spectrum of CsPbBr_3−_ *_x_*I*_x_* IPNCs and slight blue shift of the absorption spectrum of the CH_3_NH_3_PbI_3−_ *_x_*Br*_x_* layer.^27^, ^28^

According to previous study, the uniformly distributed nanocrystals allow for proper control of perovskite film growth in terms of nucleation rate and new phase formation.^27^ In this work, the IPNCs act as seed crystal toward the formation of uniform vapor‐deposited CH_3_NH_3_PbI_3_ films on cp‐TiO_2_/CsPbBr_3_ substrate by the AFM images (Figure S5, Supporting Information). Figure 2e,f depicts the cross‐sectional SEM images of the two optimized devices, which clearly show each layer. The thicknesses of each constituent layer in the PSCs are measured to be 45, 110, and 470 nm for TiO_2_, CsPbBr_3_, and CH_3_NH_3_PbI_3_, respectively. In comparison with the unmodified devices, the CsPbBr_3_ modification could enhance the light absorption of hybrid CH_3_NH_3_PbI_3_ films.

**Figure 3:**
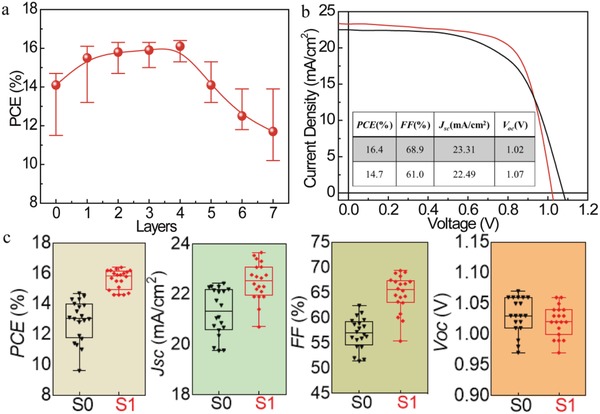
a) Device performance of the optimized devices with different layers of CsPbBr_3_ IPNCs. b) *J*–*V* curves of the device with and without IPNCs modification measured under simulated AM1.5 sunlight of 100 mW cm^−2^. c) Photoelectric parameters represented for 20 data points as a standard box plot.

In order to study whether the CsPbBr_3_ modification is beneficial to the device performance, the devices with structure of FTO/TiO_2_/CsPbBr_3_/CH_3_NH_3_PbI_3_/Spiro‐OMeTAD/Au were fabricated and characterized. The current–voltage (*J−V*) curves of the PSCs with different layers of CsPbBr_3_ are presented in **Figure** **3**a to elucidate the influence of layer thickness on photovoltaic performance. The control PSCs present a highest PCE value of 14.7% in this work. It can be seen that the PCE values exhibit a significant increase as the IPNCs spin‐coating time increases from one to four. PCE values decrease with further increasing times of spin‐coating. Although thick CsPbBr_3_ layer may provide higher contact area for CH_3_NH_3_PbI_3_, the excessive CsPbBr_3_ will agglomerate on TiO_2_ electron transport layer, affecting the crystallization of hybrid perovskite layer and leading to the decreased device performance. This can be confirmed from Figure S6 in the Supporting Information, which reflects the influence of CsPbBr_3_ with different thicknesses on the absorption spectra of CH_3_NH_3_PbI_3_ thin film. When the spin‐coating times are less than four, the absorption intensity of CH_3_NH_3_PbI_3_ films increases in the full spectrum range, which indicates the improved crystallization. In addition, significant enhancement in the range of 200–500 nm can be observed, which is attributed to the enhanced absorption of IPNCs in blue and UV region. As the IPNCs spin‐coating times is more than five, the overall absorption of the device decreases, which can be explained by the poor crystallinity of hybrid perovskite films. As a result, an optimized thickness of CsPbBr_3_ layer is about ≈110 nm, leading to the best performance with a maximum PCE of 16.4% in this work, with enhanced factor of 11.6% compared to control device. To the best of our knowledge, this is the most efficient enhanced factor in PSCs compared to previous works, which is applied to the downshifting layer inside of PSCs.^29^, ^30^, ^31^, ^32^, ^33^ The *J*–*V* curves of the optimized devices fabricated with and without CsPbBr_3_ are shown in Figure 3b. Photoelectric parameters are summarized in the inset of Figure 3b. The champion device exhibits a short‐circuit current density (*J*_sc_) of 23.31 mA cm^−2^ and fill factor (FF) of 68.9%, compared to those of control device (22.49 mA cm^−2^ and 61.0%). These results indicate that the CsPbBr_3_ layer has a significant effect on *J_sc_*, FF, and PCE. The open‐circuit voltage (*V*_oc_) of the CsPbBr_3_‐modified device is 1.02 V, which is lower than that of control devices (1.07 V). This can be related to changes of the bandgap in CH_3_NH_3_PbI_3_ after Br^−^ (CsPbBr_3_) passivation effect.^34^ 20 devices were fabricated for each condition (with and without CsPbBr_3_ IPNCs), and the photovoltaic parameters are summarized in Figure 3c. The improvement of PCE, *J*_sc_, and FF compared to those of the control devices can be attributed to lower series resistance. The narrow PCE distribution of the modified devices in Figure S7 in the Supporting Information further indicates that modified devices present excellent reproducibility compared to control devices.

**Figure 4:**
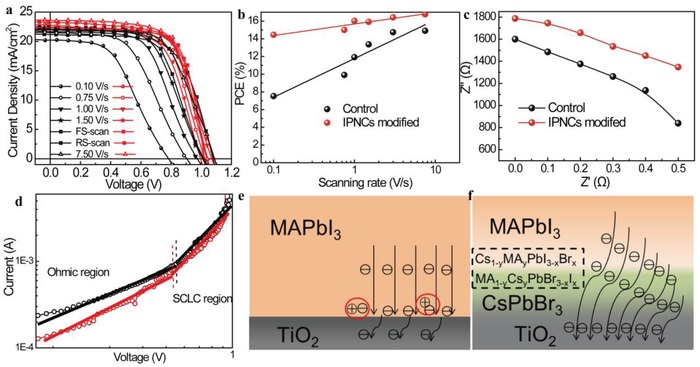
a) *J*–*V* curves of the control and IPNCs‐modified devices at different scanning rate and form the forward and reverse scan directions. b) The summed PCE values at different scanning rates. c) Calculated *R*_rec_ values from Nyquist plots of devices measured at 0–0.5 bias voltage. d) The measured electron conductivity of films from the current–voltage traces of the devices with a structure of Ag/PCBM/CH_3_NH_3_PbI_3_/(CsPbBr_3_)/TiO_2_/FTO model (n‐type) under illumination. The tested active area was confined to be 0.1 cm^2^ (0.2 cm × 0.5 cm), the thickness can be estimated to be 420 × 10^−7^ cm. Electron transport characteristics from the mechanism diagram of e) control and f) CsPbBr_3_‐modified devices.

Previous results indicate the hysteresis effect of PSCs is clearly sensitive to interface between perovskite and electron transport layer.^2^, ^35^ To study the hysteresis behavior, we characterize the device performance with different scan rates (0.1 to 7.5 V s^−1^), as shown in **Figure** **4**a. It can be seen that the control devices are strongly dependent on the scanning rate, while the IPNCs‐modified devices are less affected by the scan rates. PCE values as a function of scan rates are summarized in Figure 4b. The calculated slope of the PCE data points of the modified devices is 1.30, which is much lower than that of control device (4.37). Although the origins of hysteresis in PSCs are not well understood, one contributing factor to hysteresis believed to be inefficient is the charge extraction from the carrier layer.^36^, ^37^ The presence of CsPbBr_3_ IPNCs could achieve perfect interfacial contact, facilitating the photo‐generated electron transfer by creating electrostatic interaction between TiO_2_ and CH_3_NH_3_PbI_3_.^38^

In order to further verify the effect of interfacial engineering on carrier transmission and hysteresis effect. The recombination resistances (*R*_rec_) was studied with impedance spectroscopy (IS) measurement by fitting the results using an equivalent circuit model of *R*_s_(*R*_rec_ *C*_rec_) in Figure S8 in the Supporting Information. Accordingly, the data at middle frequency in IS patterns are related to charge recombination processes. A higher *R*_rec_ indicates a lower recombination rate, and could result in lower carrier recombination in PSC devices.^22^ *R*_rec_ values obtained with different applied voltages are shown in Figure S9 in the Supporting Information and summarized in Figure 4c. From the results, it is clearly observed that the control devices possess a low *R*_rec_, while the CsPbBr_3_‐modified devices exhibit higher *R*_rec_ at 0–0.5 V bias. By introducing IPNCs in PSCs, higher *R*_rec_ values indicate the enhanced carrier transient behaviors due to more efficient pathways for electron transport from CH_3_NH_3_PbI_3_ to TiO_2_ induced by the purified CsPbBr_3_, which causes the elimination of hysteresis effect and enhancement of FF.

We then fabricated electron‐only transporting devices (FTO/TiO_2_/CsPbBr_3_/CH_3_NH_3_PbI_3_/PCBM/Ag) to measure the electron trap‐state density to further study the enhanced transient carrier behaviors. Figure 4d shows the current–voltage traces of the devices. The linear relation indicates an ohmic response of the devices as the bias voltage is below 0.6 V, and the current increases quickly and nonlinearly when the bias voltage exceeds the kink point, demonstrating that the trap states are completely filled.^22^ The trap‐state density can be determined by the trap‐filled limit voltage (*V*_TFL_) using the equation

(1) $$V_{\mathrm{TFL}}=\frac{L^{2}e\;n_{t}}{2\varepsilon \;\varepsilon_{0}}$$

where *n*_t_ is the trap‐state density, *e* is the elementary charge, *L* is the thickness of CH_3_NH_3_PbI_3_ (450 nm), ε is the relative dielectric constant of CH_3_NH_3_PbI_3_ (ε = 32),^39^, ^40^ and ε_0_ is the vacuum permittivity. The *V*_TFL_ values of the control‐ and IPNCs‐based devices are 0.50 and 0.48 V, respectively. Therefore, the electron trap‐state density can be reduced by IPNCs modification.^35^ The CsPbBr_3_‐modified CH_3_NH_3_PbI_3_ films exhibit higher conductivities than that of control films. At space‐charge limited current (SCLC) region, the modified device exhibits higher electron mobility. The obtained results are 3.07 × 10^−4^ and 2.36 × 10^−3^ S m^−1^ for the modified and control devices, respectively, which are comparable to the previous reports.^41^ The curves in Figure 4d clearly reveal that the modification of CsPbBr_3_ IPNCs can benefit carrier transport between TiO_2_ electron transport layer and CH_3_NH_3_PbI_3_.

Above all, electron trap‐state density measurement further confirms that the high electron extraction efficiency originated from improved photoelectrical properties induced by IPNCs modification. As an efficient scaffold layer, the CsPbBr_3_ IPNCs modification could result in long‐range carrier distance and favorable ionic transport within devices.^42^, ^43^ The enhanced carrier transportation could help eliminate the hysteresis of PSCs.^44^ The possible reasons of the decreased hysteresis for PSCs can be concluded as follows: the interfacial optoelectronic properties between TiO_2_ and CH_3_NH_3_PbI_3_ were optimized by CsPbBr_3_ modification, which greatly assists the photo‐induced carrier generation and transport. In addition, IPNCs exhibit higher electrical conductivity compared to hybrid CH_3_NH_3_PbI_3_ perovskite materials. In order to further prove the role of CsPbBr_3_ IPNCs in device operation, energy dispersive X‐ray (EDX) element analysis of cross‐sectional CsPbBr_3_/CH_3_NH_3_PbI_3_ films (Figure S10, Supporting Information) was carried out and is depicted in Figure S11 in the Supporting Information. For the initial cross‐section EDX mapping, it can be seen that the main distribution thickness of Cs element is about 100 nm, with Br element distributed around Cs element. This indicates that the exchange of anion Br is faster than that of cationic Cs. After two hours, the EDX mapping from the same cross‐sectional SEM interface shows that the distribution of Cs element is widened to about 145 nm, indicating that Cs ions not only existed in CsPbBr_3_, but also entered into CH_3_NH_3_PbI_3_. At the same time, the distribution range of Br element further increased to some extent. It shows that some Br ions also enter into CH_3_NH_3_PbI_3_. The EDX mapping results indicate the ion exchange does occur between inorganic CsPbBr_3_ and hybrid CH_3_NH_3_PbI_3_ perovskite, leading to Cs_1−_ *_y_*MA*_y_*PbI_3−_ *_x_*Br*_x_* alloy between CH_3_NH_3_PbI_3_ and CsPbBr_3_ by the exchange mechanism of anions and cations. The possible mechanism diagram is shown in Figure 4e,f. Acting as a seed layer for the growth of complex composition perovskites, the CsPbBr_3_ can effectively promote electron injection from CH_3_NH_3_PbI_3_ to cp‐TiO_2_ and reduce the recombination and quenching at the interface of CH_3_NH_3_PbI_3_/TiO_2_. According to previous reports, a small amount of Cs and Br can promote the crystallization of perovskite, leading eventually to improved optical and electrical properties of complex perovskite photoactive material.^45^, ^46^ Finally, the complex perovskite behave better and lead to the improved solar cells. Previous studies indicated that optimization of electron transport layer could significantly decrease trap states and consequently reduce *J*–*V* hysteresis for the fewer capacitance elements in the traps.^36^, ^47^ Our results further prove that the origin of the better electron transport from CH_3_NH_3_PbI_3_ to TiO_2_ layer can be enhanced, while the hysteresis effect can be reduced after CsPbBr_3_ IPNCs modification due to the decreased trap states and increased electron conductivity.

**Figure 5:**
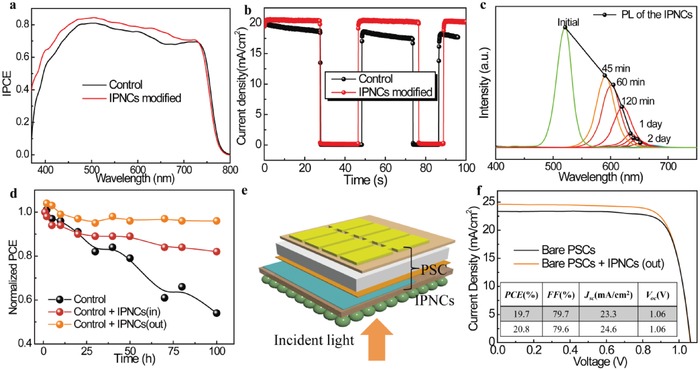
a) The IPCE result for the device with and without IPNCs modification. b) The steady‐state output of the champion device with and without IPNCs modification at a given constant bias related to the maximal power point. c) The luminescence of the IPNCs‐modified devices that vary as a function of time. d) Variation of the normalized PCE with time was obtained from *J*–*V* measurements under UV illumination. e) Device structure of the PSCs‐coated with CsPbBr_3_@SiO_2_ outside of the device. f) *J*–*V* characteristics of the bare device and CsPbBr_3_@SiO_2_‐coated device.

**Figure** **5**a displays incident photon‐to‐current conversion efficiency (IPCE) curves of the PSCs with and without IPNCs modification. As expected, obvious enhancement in IPCE is observed for the CsPbBr_3_ IPNCs‐modified device in the whole region from 400 to 800 nm in comparison with that of the control device. Notably, the modified devices show higher intensity in the blue and UV region (300−500 nm), which is ascribed to the increased photo response contributed by CsPbBr_3_ nanocrystals by photon downshifting effect.

To study the photocurrent stability, the photocurrent densities of the champion devices were recorded as a function of time when the cells were biased at their respective maximum power point values, as shown in Figure 5b.^48^ The steady‐state output of the modified device can reach a photocurrent density of 22.60 mA cm^−2^, close to the value from *J*–*V* curve. Thus, the excellent performance with reduced hysteresis achieved in the modified PSCs is attributed to the efficient carrier transport in comparison with that in the control PSCs.^21^, ^22^ The “colorful” phenomenon in solar cells devices can be observed due to the luminescence properties of IPNCs. The detailed time points for different photoluminescence intensity from 520 to 640 nm are recorded in Figure 5c. It can be seen that the layers could exhibit a photoluminescence peak at 590 nm after 45 min film fabrication. As the time increases to 2 days, the fluorescence wavelength can be extended to 650 nm with the decreased fluorescence intensity, indicating the ion exchange effect between CsPbBr_3_ and CH_3_NH_3_PbI_3_ and transformation from CsPbBr_3_ nanocrystals to MA_y_Cs_1−_ *_y_*PbBr_3−_ *_x_*I*_x_* films. We also tested the influence of CsPbBr_3_ layer on device stability under continuous 365 nm UV light illumination for 100 h in nitrogen environment. In this UV irridiation aging test, the unmodified device loses 45% of its initial PCE, dropping from 14.7% to 8.2% in Figure 5d. The CsPbBr_3_‐modified device demonstrates excellent stability under the same condition, retaining 82% of its initial PCE. This indicates that the device fabricated with IPNCs demonstrates excellent light stability. We also fabricated the device by coating IPNCs outside of the device by modify CsPbBr_3_ using silane surface coating method. Figure S12 in the Supporting Information shows the TEM image of CsPbBr_3_@SiO_2_ nanocomposite. According to previous work, silane surface coating can effectively improve environmental stability of IPNCs (CsPbBr_3_@SiO_2_ nanocomposites).^49^, ^50^ The fluorescence quantum efficiency of CsPbBr_3_@SiO_2_ nanocomposites were measured to be 79.4%, which is slightly lower than that of pure CsPbBr_3_ nanocrystals (82.8%). The air‐stable CsPbBr_3_@SiO_2_ layer was coated on the front side (transparent side) of the device as shown in the schematic diagram for estimating the influence of UV blocking for the devices(Figure 5e). The IPNCs‐coated device exhibits a PCE of 15.3%, lower than that of IPNCs‐modified devices inside. The effective PCE enhancement is mainly attributed to the photon downshifting effect of IPNCs only as IPNCs are coated outside of the device. The parameters including PCE, *J*_sc_, *V*_oc_, and FF for control, CsPbBr_3_‐modified and CsPbBr_3_@SiO_2_‐coated devices are listed in Figure S13 in the Supporting Information. The air‐stable CsPbBr_3_@SiO_2_‐coated PSCs devices could maintain higher *J*_sc_ values, while the control devices exhibit continually degraded tendency, indicating the effective UV blocking results. The CsPbBr_3_‐modified PSCs could exhibits higher FF and *V*_oc_ values than that of control devices and CsPbBr_3_@SiO_2_‐coated PSCs, revealing the efficient electrical performance improvement, mainly including the separation and transmission of photo‐generated carriers. In this UV light exposure aging test, IPNCs‐coated device loses minimum 6% of its initial efficiency under UV radiation for 100 h. The stability improvement of the coated device is more obvious than that of the CsPbBr_3_‐modified device. It is mainly attributed to the decreased fluorescence intensity and fluorescence quantum yield of IPNCs due to ion exchange as integrated inside the device (Figure 5c).^51^ In order to avoid ion exchange, we also tried to introduce CsPbBr_3_@SiO_2_ inside of PSC devices. As the CsPbBr_3_@SiO_2_ nanocomposites were introduced, the PCE dropped sharply to 6.7% (Figure S14, Supporting Information), which is mainly attributed to the carrier barrier effect of dielectric silicon dioxide on the surface of IPNCs.

To study the effect of IPNCs coating on the light stability under long time irradiation, the device with an excellent PCE of 19.7% is prepared by a typical one‐step spin‐coating CH_3_NH_3_PbI_3_ precursor method. From the *J*–*V* measurements of the PSCs‐coated outside by IPNCs in comparison with control device in Figure 5f, the *V*_oc_ and FF values are almost the same, 1.06 V and 79.7%/79.6%, indicating that the effect of IPNCs coating on *V*_oc_ and FF values of the devices is negligible. The *J*_sc_ exhibits an increase from 23.3 to 24.6 mA cm^−2^, as the optimized spin‐coating times is six. The enhanced *J*_sc_ results from the enhanced absorption by the photon downshifting effect of UV light to visible light by CsPbBr_3_@SiO_2_ nanocomposite layer. The PCE exhibits the same tendency as *J*_sc_, and increases from 19.7% to 20.8%, with the amplification of 5.6% compared to the bare device, indicating that the enhanced performance from photon downshifting effect is about 5.6%. The external quantum efficiency (EQE) spectra from 360 to850 nm further indicate that CsPbBr_3_@SiO_2_ nanocomposite–coated devices could exhibit the improved light harvesting effects than that of the bare device, especially in the region of UV and blue light as depicted in Figure S15 in the Supporting Information. The integrated photocurrent densities are 22.08 and 23.0 mA cm^−2^ (Figure S16, Supporting Information), with a 4.2% enhancement. The mismatched value inferred from the *J*–*V* curves (Figure 5f) was estimated to be ≈1 mA cm^−2^, consistent with typical reports in the PSCs field.^9^ The relatively lower integrated photocurrent densities than that of measurement result can be attributed to the unmeasurable range below 360 nm and integral error.^52^ The results are in agreement with the tendency of *J*_sc_ derived from the *J*−*V* measurement and previous results by photon downshifting effect.^9^, ^53^ As compared, the IPNCs inside modified PSCs exhibit an improvement of 11.6%, indicating the non‐negligible role of carrier transportation for the effective modification. Under UV illumination for 100 h, the CsPbBr_3_‐coated device retains about 96% of its initial PCE, from 20.8% to 19.9%. The photovoltaic result shows that, as a photo downshifting layer, IPNCs can effectively improve the light response of the PSCs in UV region when IPNCs are placed either inside or outside the device. However, much better device performance is obtained as IPNCs are incorporated inside the device due to the excellent carrier transmission characteristics and higher electrical conductivity induced by IPNCs. The light stability of the bare and air‐stable CsPbBr_3_@SiO_2_‐coated PSCs were also studied. Both of the PSCs were stored in ambient atmosphere at room temperature without encapsulation and the corresponding PCEs changes were plotted in Figure S17 in the Supporting Information. The *J*–*V* curves were recorded at intervals under AM 1.5G illumination intensity. It can be found that the CsPbBr_3_@SiO_2_‐coated PSCs device could retainan average of 92% values (maximum at 98%) of its initial PCE after 1000 h irradiation under simulated light, while the bare device exhibits a dramatically decreased PCE to 64%. This stability result further proves the photon downshifting effect of CsPbBr_3_ and its blocking effect on UV light, which is beneficial to improve the light stability of the device. Here, it can be concluded that the CsPbBr_3_ between TiO_2_ and CH_3_NH_3_PbI_3_ play three important roles. First, at the interface between CH_3_NH_3_PbI_3_ and CsPbBr_3_ layers, the electron transfer process from CH_3_NH_3_PbI_3_ to TiO_2_ electron transport layer is improved, leading to increased PCE. Second, light harvesting in UV region is enhanced via photon downshifting effect of IPNCs to improved PCE. Third, IPNCs as a UV filter can significantly retard the UV photo‐induced degradation of hybrid perovskite films to increase light stability.

## 3 Conclusions

In this work, we present a study of the application of CsPbBr_3_ IPNCs to improve the photovoltaic performance and light stability of PSCs by enhanced carrier transport and photon downshifting effect. Incorporating IPNCs layer (≈110 nm) leads to the device with a maximum PCE of ≈16.4%, with the amplification of 11.6% compared to the control devices. To the best of our knowledge, this is the first report about improving the PCE and stability of PSCs with the introduction of CsPbX_3_ nanocrystals inside the devices. This strategy could exhibit the highest enhanced factor of 11.6% compared with the traditional photon downshifting material. Additionally, the outside CsPbBr_3_@SiO_2_ coating could increase the PCE from 19.7% to 20.8%, which is improved by 5.6% compared to the uncoated device. More importantly, CsPbBr_3_ IPNCs‐modified PSCs exhibit much better stability under UV light irradiation compared to the control devices. The fundamental mechanism in optical and electrical properties reveals that the modification of IPNCs can provide noticeable advantages, including enhanced light harvesting and the electron injection from CH_3_NH_3_PbI_3_ hybrid perovskite layer to TiO_2_ electron transport layer. Furthermore, integrating IPNCs inside could induce specific color fluorescence and colorful perovskite devices under UV illumination. The IPNCs modification developed in this work exhibits significant potential toward the fabrication of PSCs with high PCEs.

## 4 Experimental Section

*Materials and Precursor Preparation*: Chemicals for preparation like Cesium bromide (CsBr, 99.9%), Cesium iodide (CsI, 99.9%), lead diiodide (PbI_2_, 99.9985%), lead dibromide (PbBr_2_, 99.999%), and Cesium carbonate (CsCO_3_, 99.9%) were obtained from the Alfa Aesar. Hydroiodic Acid (HI, 57%) and Hydrobromic acid (HBr, 48%) were obtained from Aladdin Reagents Co., Ltd. (Shanghai, China). Oleylamine (OA, 98%), and Octa‐decene (ODE, 98.0%) were purchased from MACKLIN Co., Ltd. (China). Spiro‐OMeTAD powders were purchased from Xi'an Polymer Light Technology Corp (China). Spiro‐OMeTAD hole transporting solutions were made by dissolving 72.3 mg of Spiro‐OMeTAD powders (Xi'an Polymer Light Technology Corp, China) in 1 mL of chlorobenzene (CB) and stirring it at 70 °C for 2 h. Then, 17.5 µL of Li‐bis(trifluoromethanesulfonyl) imide (Li‐TFSI) (520 mg mL^−1^ in acetonitrile) and 28.3 µL of 4‐tert‐butylpyridine (t‐BP) were added to the solution in a nitrogen‐filled glove box and stirred for 30 min until used for coating. The other materials and chemicals were purchased from MACKLIN Co., Ltd (China) and all chemicals were used as received. The CsPbBr_3_ IPNCs was prepared according to the previous reports. Specifically, the obtained precipitates were first washed with toluene through centrifugation at 9800 rpm for 10 min. Then the precipitates were further washed with ethyl acetate through centrifugation at 9000 rpm for 1 min. This washing process was repeated for 4 times to eliminate organic ligands on the surface as much as possible. Finally, the precipitates were redispersed in ethyl acetate and formed a stable colloidal solution.

*Device Fabrication and Characterization*: All device fabrication was carried out in glove. FTO/glass substrate was etched with 30% HCl and zinc powder was removed for anode contact. Then the substrates were cleaned with detergent (Youxuan Technology, Liaoning, China), deionized water, ethanol, and acetone. Finally they were treated under oxygen plasma for 15 min to remove organic residues. Cp‐TiO_2_ layers were deposited by spin‐coating of titanium (IV) isopropoxide (3.24 g) in butanol (50 mL) solution with a speed of 2000 rpm. After that, the substrates with titanium (IV) isopropoxide were annealed in air at 500 °C for 30 min. PbI_2_ and CH_3_NH_3_I powders were used to fabricate CH_3_NH_3_PbI_3_ films via thermal evaporation process. The evaporation current for PbI_2_ was 32 A by the inorganic vapor source with evaporation rate of 0.24 A s^−1^, and CH_3_NH_3_I was evaporated by organic vapor source at 320 °C with evaporation rate of 0.31 A s^−1^. The chamber pressure was controlled to be ≈6.4 × 10^−4^ Pa. The film thickness was detected by the film‐thickness meter in the chamber and controlled by the evaporation time. For the one‐step perovskite base devices preparation, PbI_2_ (462 mg) and CH_3_NH_3_I (163 mg) were first dissolved in mixed solvent (DMSO:DMF = 3:7, v: v) and stirred at 60 °C for 4 h before use. Then 100 µL mixture was deposited onto the FTO/cp‐TiO_2_ by a successive spin‐coated process of two steps, i.e 6000 rpm for 9 s and 4000 rpm for 40 s to form CH_3_NH_3_PbI_3_ film. Specifically, 600 mL diethyl ether was dropped on the surface. The second spin‐coating process was for 15 s. Then the antisolvent‐treated CH_3_NH_3_PbI_3_ films were dried at 100 °C for 10 min.

The solar cell measurement and storage were performed in ambient conditions, without any encapsulation. The *J*–*V* curves of the PSCs were obtained by a solar simulator (ABET Sun 2000) at AM1.5 and 100 mW cm^−2^ illumination calibrated with a reference silicon cell (RERA Solutions RR‐1002) using a Keithley 2400 as a source‐meter. IPCE was recorded by using SolarCellScan100 as a function of wavelength from 380 to 800 nm. The spectral EQE responses were taken from 360 to 850 nm by an EQE measurement system (QEX10, PV measurement). The surface morphology of the films was characterized by a Sirion field‐emission scanning electron microscope. XRD measurements for the prepared films was carried out on a Rigaku D/max 2550 X‐ray diffractometer using a monochromatized Cu target radiation source at a scanning rate of 18° min^−1^. The electrical conductivity was measured by a Keithley 2400 as a source‐meter, with current–voltage traces from +3 V to −3 V.

## Conflict of Interest

The authors declare no conflict of interest.

## Supporting information

Supplementary
