# Supplementary material for: Interfacial Engineering and Photon Downshifting of CsPbBr3 Nanocrystals for Efficient, Stable, and Colorful Vapor Phase Perovskite Solar Cells
Source: Adv Sci (Weinh). 2019 Apr 20;6(11):1802046. doi: 10.1002/advs.201802046 (PMC6548969; doi:10.1002/advs.201802046)
Supplement: Supplementary file 1 — Supplementary [file ADVS-6-1802046-s001.pdf]

Copyright WILEY-VCH Verlag GmbH & Co. KGaA, 69469 Weinheim, Germany, 2019.

ADVANCED SCIENCE
Open Access

Supporting Information

for *Adv. Sci.,* DOI: 10.1002/advs.201802046

Interfacial Engineering and Photon Downshifting of $CsPbBr_3$ Nanocrystals for Efficient, Stable, and Colorful Vapor Phase Perovskite Solar Cells

Cong Chen, Yanjie Wu, Le Liu, Yanbo Gao, Xinfu Chen, Wenbo Bi, Xu Chen, Dali Liu, Qilin Dai,* and Hongwei Song**

Supporting information

Interfacial Engineering and photon downshifting of $CsPbBr_3$ nanocrystals for efficient, stable and colorful vapor phase perovskite solar cells

Cong Chen, Yanjie Wu, Le Liu, Yanbo Gao, Xinfu Chen, Wenbo Bi, Xu Chen, Dali Liu*, Qilin Dai* and Hongwei Song*

Dr. C. Chen, Dr. Y. Wu, Dr. L. Liu, Dr. Y. Gao, Dr. X. Chen, Dr. W. Bi, Dr. X. Chen, Prof. D. Liu, Prof. H. Song
State Key Laboratory on Integrated Optoelectronics, College of Electronic Science and Engineering, Jilin University, 2699 Qianjin Street, Changchun, 130012, People's Republic of China.
E-mail: ldl@jlu.edu.cn, songhw@jlu.edu.cn

Prof. Q. Dai
Department of Chemistry, Physics, and Atmospheric Sciences, Jackson State University, Jackson, Mississippi 39217, USA.
E-mail: qilin.dai@jsums.edu

Keywords: perovskite solar cells, vapor phase, downshifting, stability, colorful

Figures and captions in supporting information

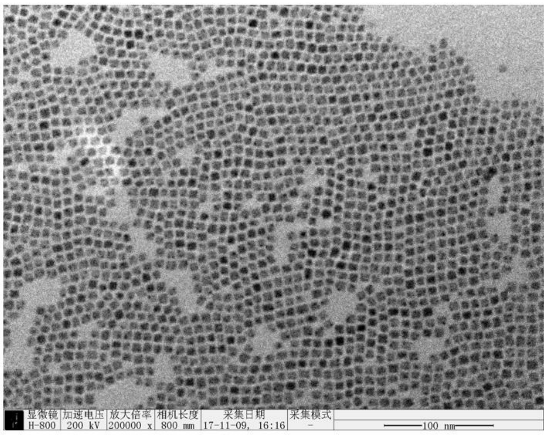

Figure S1. The TEM images of the as-prepared $CsPbBr_3$ IPNCs.

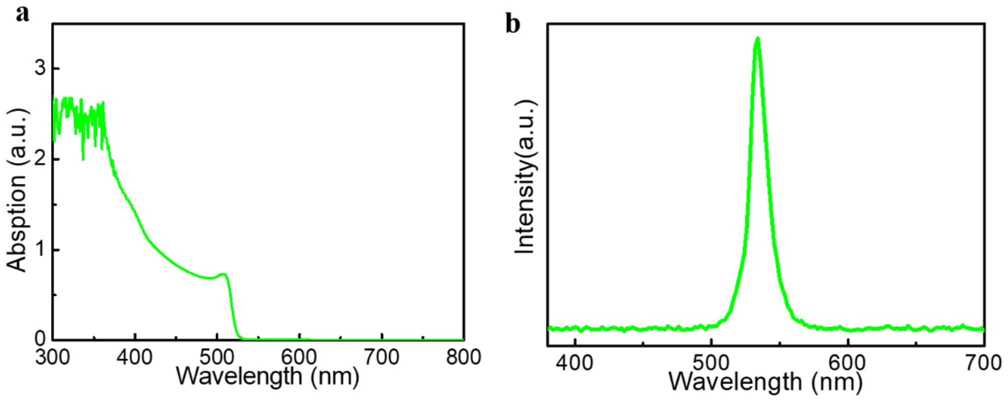

Figure S2. Absorption and emission spectra of the prepared $CsPbBr_3$ IPNCs.

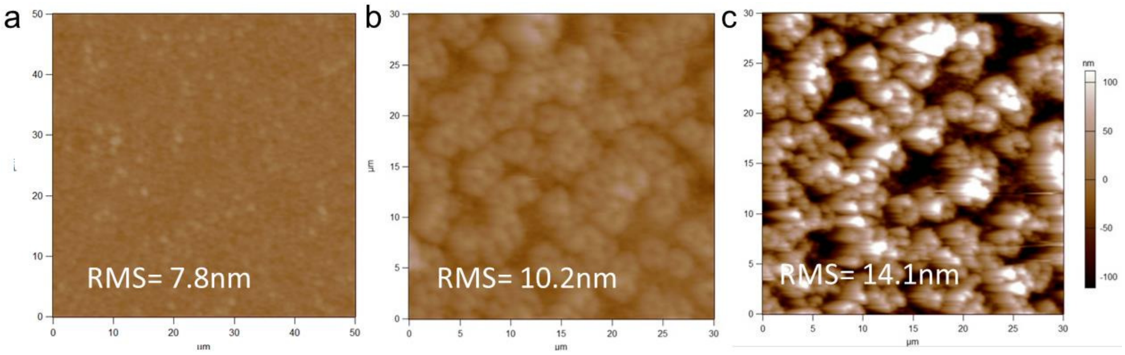

Figure S3. AFM images of the substrate with different layers of spin-coated $CsPbBr_3$ IPNCs.

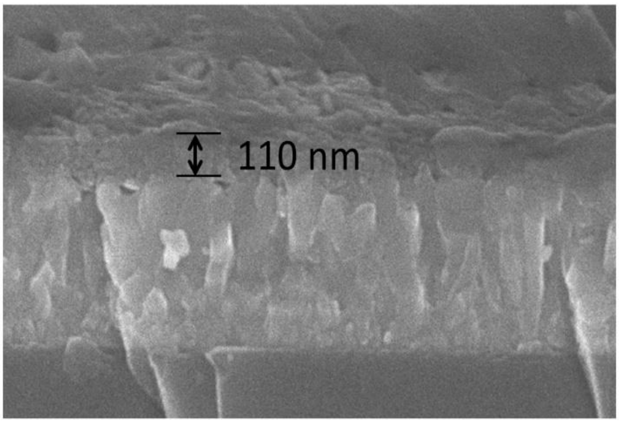

Figure S4. Cross-section SEM images of the 5 layers of IPNCs deposited on FTO/cp-TiO_2 substrate.

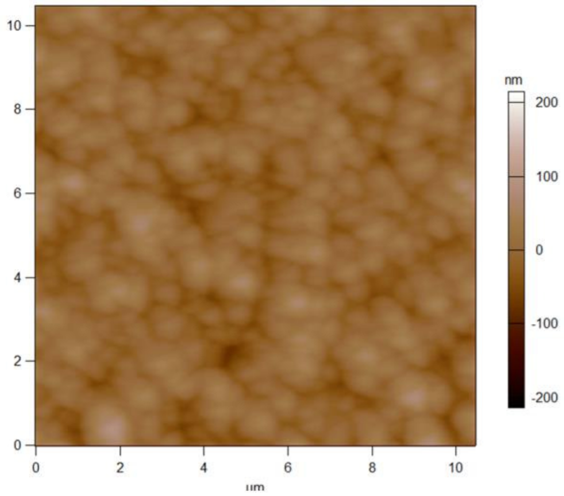

Figure S5. AFM images of the vapor deposited $CH_3NH_3PbI_3$ films on cp-TiO_2/$CsPbBr_3$ (4 layer) substrates.

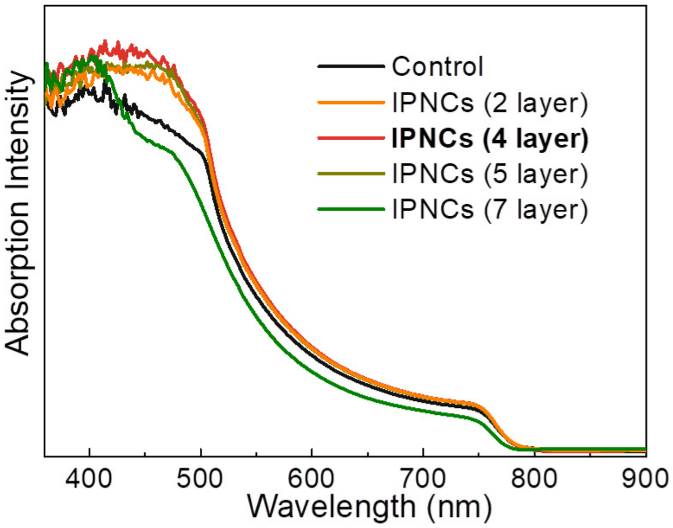

Figure S6. The UV/Vis absorption spectra $CH_3NH_3PbI_3$ samples based different layers of IPNCs.

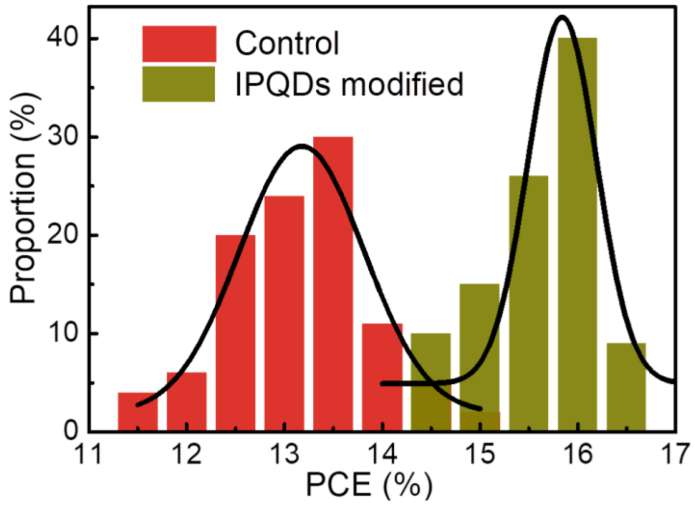

Figure S7. Statistical data of device performance for control and $CsPbBr_3$ modified PSCs. The overall proportion data are fitted by Gauss.

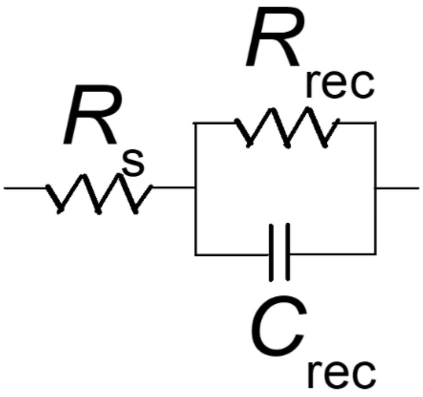

Figure S8. The equivalent circuit model of $R_s(R_{rec}C_{rec})$ for fitting the Nyquist plots in Figure S9.

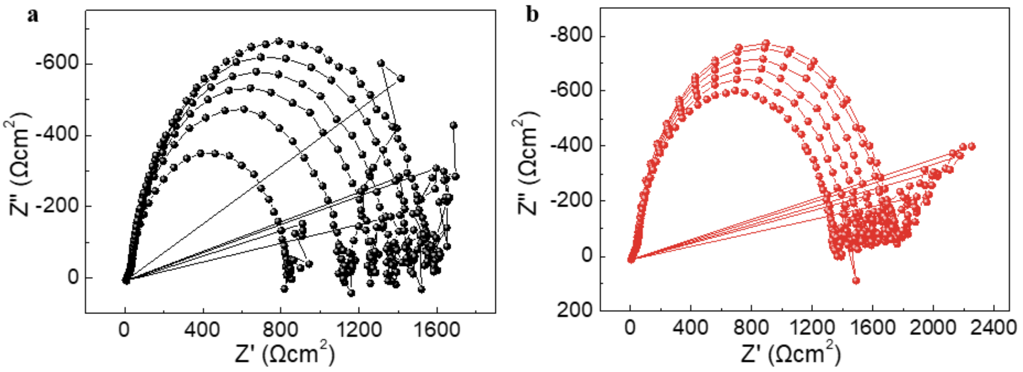

Figure S9. The Nyquist plots of control and $CPbBr_3$ modified PSCs at a frequency range from 100000 Hz to 0.1 Hz measured 10 mW/cm^2 illumination at bias voltages from 0 to 0.5 V.

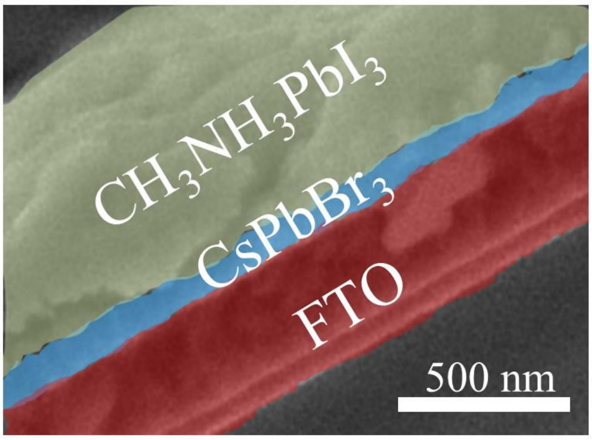

Figure S10. Cross-sectional FTO/$CsPbBr_3$/$CH_3NH_3PbI_3$ films to conduct the EDX element mapping analysis.

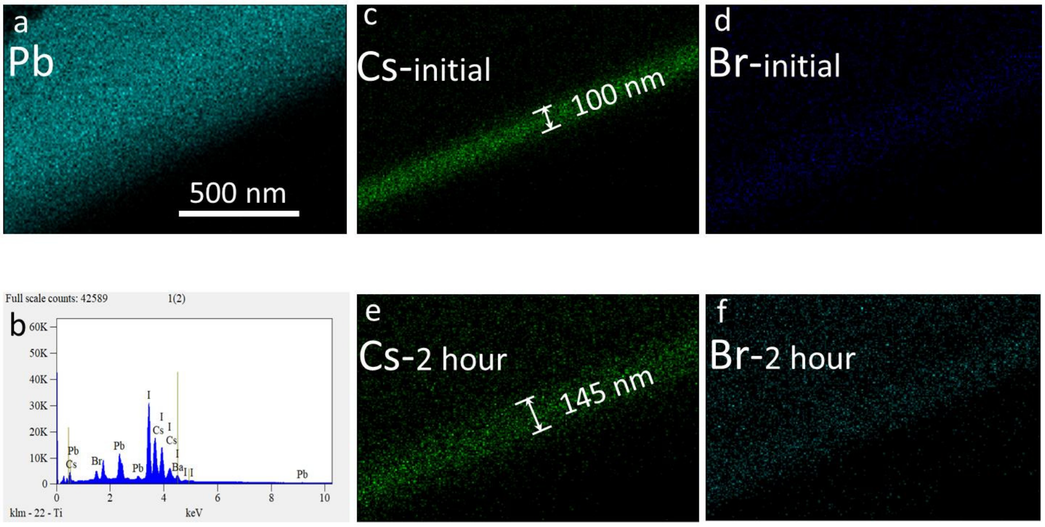

Figure S11. The EDX element mapping analysis from the cross-sectional FTO/$CsPbBr_3$/$CH_3NH_3PbI_3$ films.

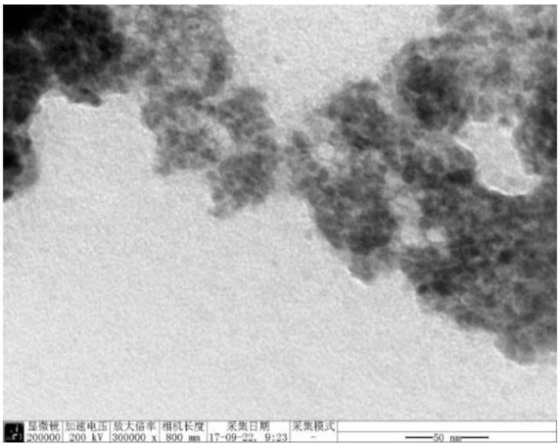

Figure S12. TEM image of the prepared $CsPbBr_3@SiO_2$ nanocomposites.

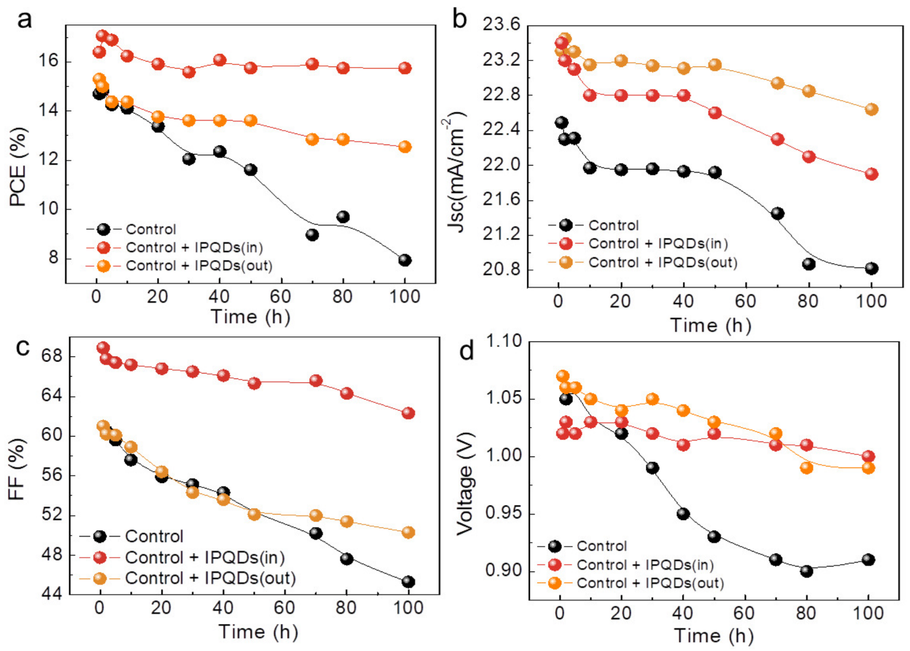

Figure S13. Device performance including PCE, J_{sc}, V_{oc} and FF parameters of the control, $CsPbBr_3$ modified and $CsPbBr_3@SiO_2$ coated devices as a function of time is shown for determining the stability performance.

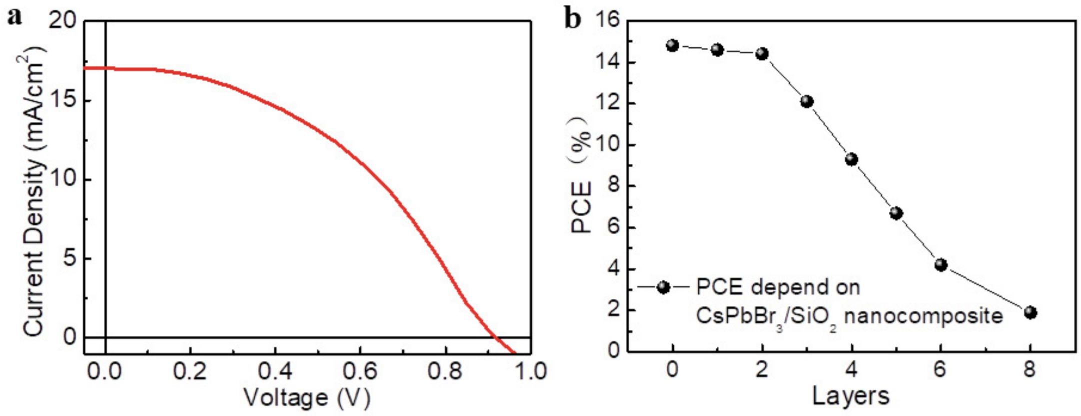

Figure S14. PCE of different layers of $CsPbBr_3@SiO_2$ nanocomposite modified PSCs. a) J-V curves of PSCs based four layers of $CsPbBr_3@SiO_2$ nanocomposites. b) PCE changing of the PSCs with different layers of $CsPbBr_3@SiO_2$ nanocomposite.

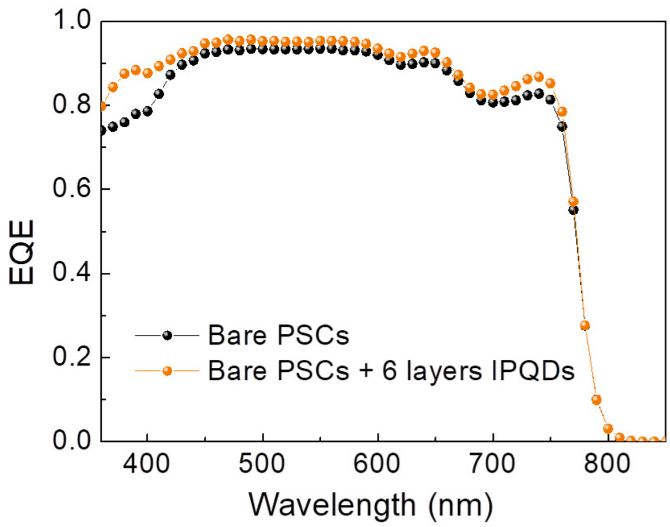

Figure S15. EQE spectra for bare and $CsPbBr_3$ coated PSCs.

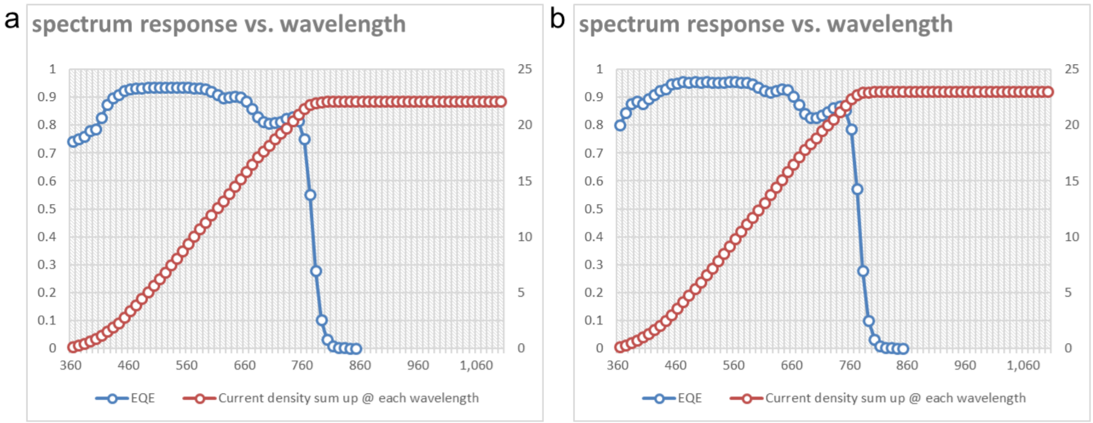

Figure S16 Integrated current values from the EQE spectrum. a) is the calculating figure for the bare device and b) for the $CsPbBr_3$ IPNCs coated PSCs.

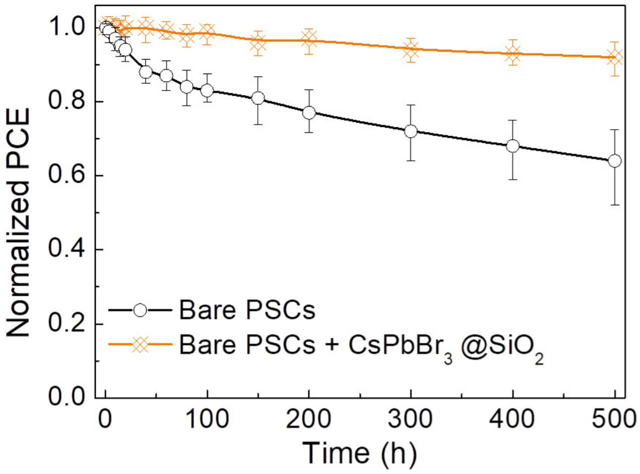

Figure S17. Normalized PCE values of the devices based on the bare and air stable $CsPbBr_3@SiO_2$ coated PSCs. The normalized PCE values with time are obtained from J–V measurements under AM 1.5G illumination.
